# New Perspectives in the Exploration of Korsakoff’s Syndrome: The Usefulness of Neurophysiological Markers

**DOI:** 10.3389/fpsyg.2016.00168

**Published:** 2016-02-16

**Authors:** Mélanie Brion, Anne-Lise Pitel, Fabien D’Hondt

**Affiliations:** ^1^Laboratory for Experimental Psychopathology, Psychological Sciences Research Institute, Université catholique de LouvainLouvain-la-Neuve, Belgium; ^2^INSERM, Unité U1077, École Pratique des Hautes Études, Université de Caen-Basse Normandie – GIP Cyceron – CHU CaenCaen, France

**Keywords:** Korsakoff’s syndrome, alcohol-dependence, electroencephalography, event-related potentials, dualprocess models, emotion, cognition

## Abstract

This perspective aims at underlining the usefulness of event-related potentials (ERP) to better understand the brain correlates of Korsakoff’s syndrome (KS), a neuropsychiatric disease characterized by severe memory impairment and most frequently resulting as a neurological complication of alcohol-dependence (AD). While ERP have been broadly used in AD, it has up to now been very little applied in KS or in the comparison of KS and AD. Within the framework of dual-process models, an influential theory postulating that addictive states result from an imbalance between under-activated reflective system and over-activated automatic-affective one, this paper proposes: (1) a brief synthesis of the main results of ERP studies in AD and KS, and (2) new research avenues using ERP to identify the electrophysiological correlates of cognitive and emotional dysfunction in KS. These experimental perspectives aim at exploring the continuity hypothesis, which postulates a gradient of impairments from AD to KS. We conclude on the possibility of developing neuropsychological strategies with electrophysiological follow-up to ensure KS diagnosis and test the efficacy of patient’s neurocognitive rehabilitation.

## Introduction

Korsakoff’s syndrome (KS), a frequent neurological complication of alcohol-dependence (AD), is mainly caused by the combined effect of thiamine deficiency and alcohol neurotoxicity. KS is classically associated with disorientation, confabulation, and amnesia (Victor et al., 1971), severe anterograde and retrograde memory deficits constituting the key symptom of KS (Butters and Cermak, 1980; Fama et al., 2012). Several studies found that KS’ brain damages and cognitive impairments are more severe than those reported in AD patients (e.g., Brokate et al., 2003; Brand, 2007; Pitel et al., 2008, 2012; Sullivan and Pfefferbaum, 2009). This has notably led to the continuity hypothesis (see Pitel et al., 2014 for a recent review; Ryback, 1971), which assumes a gradual worsening of memory deficits between “uncomplicated” AD and KS. Beyond memory impairments, recent studies have also emphasized executive (Van Oort and Kessels, 2009; Maharasingam et al., 2013) and emotional (Montagne et al., 2006; Labudda et al., 2008) dysfunctions in KS, but the continuity hypothesis has been little explored for these impairments. It has recently been proposed (Brion et al., 2015) that a dual-process perspective, which represents a well-validated conception in the addiction field (Bechara, 2005; Bechara and Damasio, 2005; Mukherjee, 2010; Noël et al., 2010), might constitute a reliable theoretical background to address this shortcoming. The dual-process models assume that every adapted human behavior (e.g., a decision-making) mobilizes the interaction between two systems: (1) the “reflective system” (mostly relying on prefrontal areas), a controlled and inhibitory process, relying on memory and executive functions to initiate controlled-deliberate responses (response-consequences link), and (2) the “automatic-affective system” (mostly relying on limbic areas), an appetitive system triggering impulsive responses based on associative learning (stimulus–response link). Actually, the automatic-affective system can be divided into an affective subcomponent associated with the core affect decoding (e.g., facial expression or prosody) and an automatic subcomponent related to the attribution of a pleasant or aversive value to environmental stimuli through conditioning (Bechara, 2005; Bechara and Damasio, 2005). According to these models, AD is characterized by a combination of weak executive functioning related to the reflective system (e.g., reduced ability to refrain drinking behavior) and inadequate automatic-affective processing (e.g., strong appetitive drive toward alcohol, emotion perception impairments, Field et al., 2010; Noël et al., 2010). As KS exploration has up to now been focused on the reflective system, it appears necessary to go beyond this classical exploration of cognitive functions to assess emotional abilities as well as emotion-cognition interactions and revisit the classical picture/description of cognitive function in KS.

**FIGURE 1:**
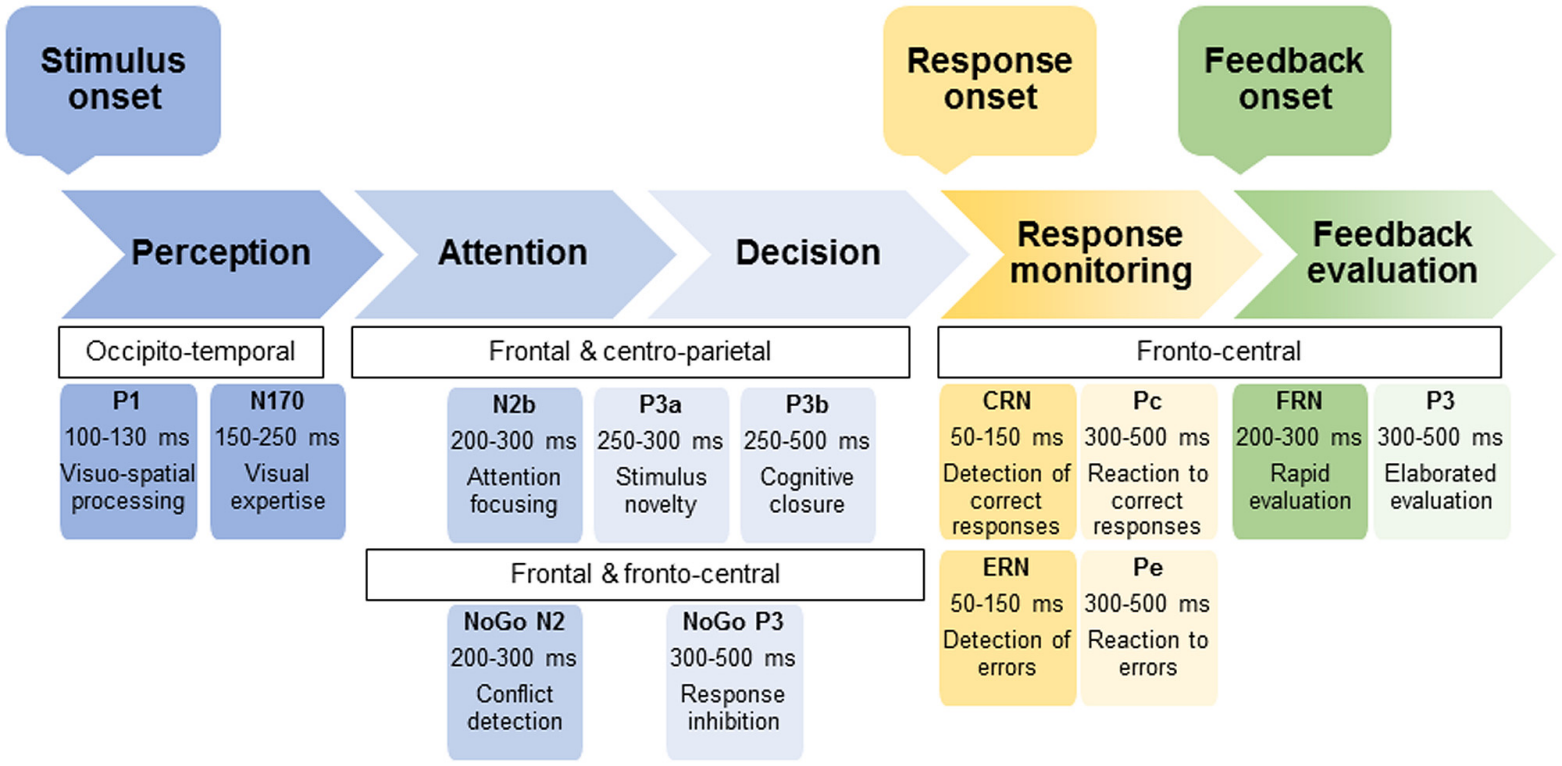
**Outline of the sequence of ERP components associated with the successive processing steps of (visual) stimulus, response, and feedback**. The name of ERP component usually begins either by P or N to indicate positive or negative voltage deflections, respectively. Particular cases are the Correct-Related Negativity (CRN) and Error-Related Negativity (ERN) as well at the Feedback-related Negativity (FRN). For each ERP, the scalp distribution, time window of the peak as well as main functional significance are provided.

An interesting way to conduct these cognitive-emotional explorations in KS is the event-related potentials (ERP) technique. Indeed, this non-invasive tool, which allows measuring brain electrical activity associated with cognitive functioning, constitutes a method of choice to assess cognitive deficits in pathological populations and has notably proven its usefulness among several psychiatric disorders (Pogarell et al., 2007; McLoughlin et al., 2014). In particular, ERP present the advantage (as compared to other neuroimaging tools like Magnetic Resonance Imaging) to have a high temporal resolution, enabling the detailed investigation of successive steps associated with stimulus processing (i.e., perceptual, attentional, and decisional stages) as well as performance and feedback monitoring [see **Figure 1** illustrating the sequence of main ERP components during (visual) information processing; Rugg and Coles, 1995; Falkenstein et al., 2000; San Martin, 2012; Walsh and Anderson, 2012; Kamarajan and Porjesz, 2015]. This method enables identifying the component related to the onset of dysfunctions, and then inferring the associated impaired processing stage (Rugg and Coles, 1995). While ERP have been fruitfully used for decades to explore brain correlates of AD, some authors highlighting the potential utility of P3 as an endophenotype of AD (Campanella et al., 2014; Kamarajan et al., 2015), only very few ERP studies have been conducted on KS. This analysis is quite surprising, considering also that ERP technique is well-suited to experimentation with patients with severe deficits like KS.

Accordingly, the aim of the present paper is twofold. First, we report the results of the few ERP studies in KS. We also report the most robust findings from studies in AD that investigated both systems postulated by dual-process models (see Kamarajan and Porjesz, 2015 for a recent review). Second, we propose new research avenues to renew the investigations of core symptoms associated with KS using ERP and to identify neurophysiological vulnerability markers of the condition. Both parts of the paper are presented through the perspective of the continuity hypothesis and the dual-process models.

## What Have ERP Studies Revealed About Reflective and Automatic-Affective Systems?

### ERP Investigations in KS

Initial ERP studies in KS were conducted in the 1980s and explored sensory processing. Several of them investigated brainstem auditory evoked responses and showed reversible abnormalities in some KS patients (Chu and Squires, 1980; Chan et al., 1985; Hammond et al., 1986). In the same vein, Chan et al. (1986) recorded visual evoked responses and found that KS patients showed delayed and reduced P1. St Clair et al. (1985) used a two-tone discrimination task and found that KS patients showed reduced amplitudes specifically for early auditory ERP (i.e., N1/P2 complex) as compared with matched non-alcoholic controls. These initial studies thus evidenced that KS is associated with impairments at the early stage of information processing. More recently, Nahum et al. (2015) recorded ERP during a continuous recognition task and showed that KS patients differed from both AD and controls as they showed a lower recognition percentage and an absence of a left medial temporal lobe dependent positivity peaking between 250 and 350 ms following immediate picture repetitions. Although these recent findings shed new light on the brain correlates associated with anterograde amnesia classically observed in KS, it has to be underlined that the current ERP data on KS remain highly scarce, especially regarding high-level processes.

### ERP Investigations in AD

Many ERP studies in AD explored the reflective system and showed that the executive dysfunctions classically found by neuropsychological studies (Field et al., 2010; Noël et al., 2007, 2012) were associated with modifications in the amplitude and/or latency of several ERP components during Go–NoGo, Stop Signal, and Flanker Tasks paradigms (see Hansenne, 2006; Kamarajan and Porjesz, 2015 for reviews). The most robust finding is probably related to the P3, with numerous studies showing reduced amplitude and increased latency of P3 in various types of task. In particular, reduced P3 amplitude has been observed in AD during NoGo trials (Cohen et al., 1997; Kamarajan et al., 2005), which can be interpreted as an altered response inhibition (Randall and Smith, 2011). Before that, conflict monitoring also appears to be altered as suggested by reduced N2 peak amplitudes for Go and NoGo conditions in AD compared with controls as well as the lack of significant increase in N2 amplitude in NoGo compared to Go trials in AD (Pandey et al., 2012; see also Donkers and van Boxtel, 2004; Randall and Smith, 2011). However, AD individuals show greater error-related negativity (ERN; Schellekens et al., 2010; Padilla et al., 2011) and correct-related negativity (CRN; Padilla et al., 2011) amplitudes compared with controls. Considering that behavioral performance is preserved, this may suggest a compensatory strategy for inhibition deficits in AD, involving enhanced performance monitoring (Padilla et al., 2011).

Regarding the affective subcomponent, results from ERP studies using an affective oddball paradigm suggest that emotional decoding deficits frequently observed at the behavioral level in AD (e.g., Philippot et al., 1999; Kornreich et al., 2001; Maurage et al., 2009a; D’Hondt et al., 2014a,b, 2015; Donadon and Osório Fde, 2014) are associated with alterations all along the information-processing stream, from early visual (delayed P100) and face-processing (delayed and reduced N170) stages to decision stage (delayed and reduced P3b; Maurage et al., 2007, 2008a). Slower early processing of emotional facial stimuli, as indexed by a delayed frontal P160, has also been observed during gender identification and emotion identification tasks in AD (Fein et al., 2010). Furthermore, using an emotional oddball paradigm with morphed stimuli, Maurage et al. (2008b) reported a specifically disrupted processing of anger (vs disgust) in AD at attentional (delayed N2b/P3a complex) and decisional (delayed and reduced P3b) stages.

Regarding the automatic subcomponent, cognitive bias toward alcohol stimuli appears to be the best predictor of relapse vulnerability (Petit et al., 2015). This bias observed at the behavioral level (e.g., Klein et al., 2013; Woud et al., 2014) may be associated with an enhanced motivational processing of alcohol-related cues as indexed by higher P3 amplitude in response to alcohol-related words (e.g., Genkina and Shostakovich, 1983; Herrmann et al., 2000; but see Hansenne et al., 2003) or pictures (Namkoong et al., 2004) compared with neutral stimuli. Thus, hyperactivity of the automatic subcomponent may explain the increased appetitive value of alcohol-related stimuli in AD, and thus poorly deliberated responses triggered by these cues. Conversely, lower P3 amplitude for incentive stimuli may reflect a reduced processing of other rewards in AD (Porjesz et al., 1987).

To sum up, although ERP studies have provided large evidence of impairments for both reflective and automatic-affective systems in AD, almost nothing is known concerning the electrophysiological correlates of systems’ dysfunctions in KS. Importantly, while imbalance between systems is considered as a critical feature of AD (e.g., Field et al., 2010), its electrophysiological correlates remain to be investigated. The main aim of the following perspective section is thus to propose new research avenues to further explore core symptoms of KS and to compare electrophysiological patterns between KS and AD by investigating both systems separately as well as their interactions.

## Toward New ERP Investigations of Reflective and Automatic-Affective Systems: From AD to KS

Beyond memory impairments, we propose that future ERP studies may determine whether there is a continuity from AD to KS in deficits regarding both systems postulated by dual-process models, and their interactions:

### Reflective System Exploration

**FIGURE 2:**
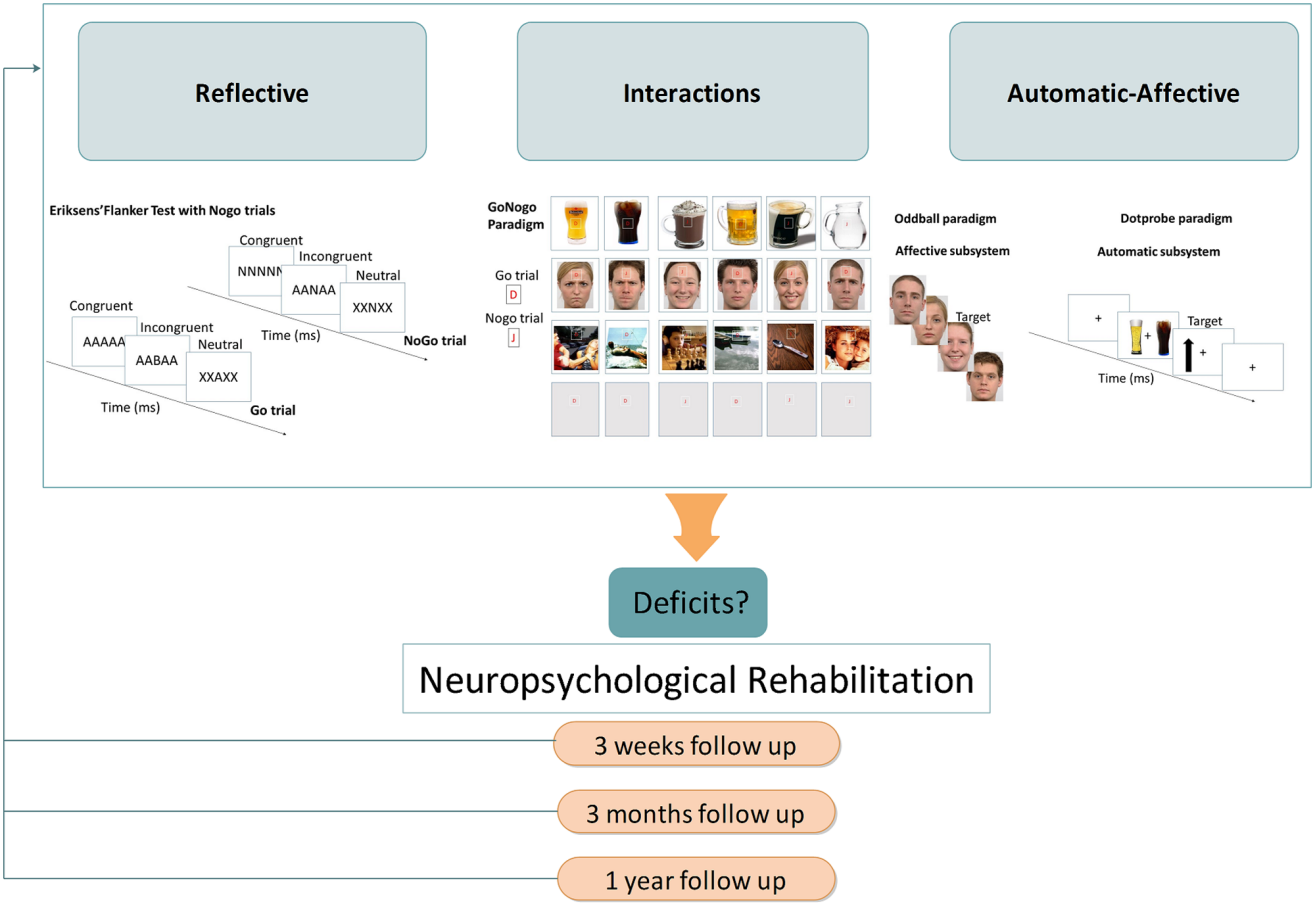
**Proposal of an ERP routine from diagnostic until neuropsychological rehabilitation among AD and KS patients within a dual-process framework**. Description of the proposed paradigms for the reflective, automatic, and affective systems as well as interactions between systems, respectively: (i) Reflective: a Flanker task with NoGo trials as proposed by Sokhadze et al. (2008) in which participants have to quickly respond to a central target flanked by distracting stimuli. (ii) Automatic: a dot-probe paradigm consisting of two stimuli (i.e., alcohol-related and neutral) that are briefly shown and followed by a probe at the location previously occupied by one of the two stimuli. The participant is required to respond as fast as possible to the probe. (iii) Affective: an emotional oddball paradigm with frequent stimuli (i.e., neutral facial expression) interrupted by deviant ones (i.e., emotional facial expression). (iv) Interactions: a Go/No-Go task using frequent Go signals (letter “D”), and rare No-Go signals (letter “J”). Both letters are superimposed on a background picture, either black, alcohol- (for the study of reflective and automatic interactions) or non-alcohol-related, neutral or emotional (for the study of reflective and affective interactions).

A gradual decline from AD to KS has been recently hypothesized for inhibition (Brion et al., 2014), given the severe impairments already described in KS (Fujiwara et al., 2008; Pitel et al., 2008). To determine the stage(s) of processing that is (are) potentially concerned by this gradual decline, it would be interesting to use an Eriksen flanker test with NoGo conditions to study response inhibition (e.g., Sokhadze et al., 2008; see **Figure 2**). This kind of modified Flanker paradigm, which allows measuring the classical stimulus-locked and response-locked ERP (see **Figure 1**), would enable determining whether there is a graduated effect of: (1) deficits in inhibition abilities from AD to KS for conflict detection (indexed by reduced NoGo N2) and/or response inhibition (indexed by reduced NoGo P3), both stages being affected in AD; (2) greater allocation of resources to performance monitoring as previously shown by response-locked ERP modifications in AD (i.e., enhanced CRN and ERN amplitudes).

### Automatic-Affective System Exploration

#### Automatic Sub-Component

How the increased “salience” attribution to alcohol-related cues and associated biases in AD (e.g., Field et al., 2009; Vollstadt-Klein et al., 2012) evolve with neurological complication remains an open issue. We propose that ERP would be particularly helpful to address at least three main questions:

(1) What is the real nature of the biases? Are they perceptual (greater salience associated with “expertise” for these cues), attentional (greater allocation of attentional resources) or decisional (less inhibition at the post-attentional stage)? Visual oddball paradigms (e.g., Petit et al., 2015) should be used to determine whether P3 modifications previously observed in AD are also present and stronger in KS, but also to investigate earlier information processing steps (i.e., perceptual and attentional stages).
(2) Do alcohol-related stimuli capture attentional resources of KS individuals? ERP recording during a dot-probe task (e.g., Bar-Haim et al., 2007) would help to determine whether AD and KS present higher P1 amplitude for targets occurring at locations previously occupied by alcohol-related cues compared to neutral ones. This has been recently observed among social drinkers low in alcohol sensitivity (Shin et al., 2010) and may reflect a potential top-down mechanism by which selective attention for alcohol cues affects early visual processing of subsequent stimuli.
(3) Is the putative hyperactivity for alcohol cues conversely associated with a decreased reactivity for non-alcohol related natural rewards (e.g., money)? Future ERP studies in KS should investigate whether the possible hyperactivity of the automatic subsystem for alcohol-related cues is accompanied by hypoactivity of this subsystem for another type of rewards, as indexed by lower P3 amplitude to incentive stimuli in AD (Porjesz et al., 1987).

#### Affective Sub-Component

While some behavioral findings suggest emotional impairments in AD and KS (see Brion et al., 2015, for a review), one important question still to be addressed in KS is the onset of dysfunction along the information-processing stream. Given results from ERP studies that showed sensory deficits in KS, future ERP studies should determine whether emotion deficits also arise as soon as visual steps, as observed in AD using an emotional oddball paradigm (Maurage et al., 2007, 2008a; see **Figure 2**), and are more severe in KS. Importantly, further ERP studies should investigate more deeply the putative deficits of visual pathways in KS and the interaction between vision and emotion as recently proposed for AD (D’Hondt et al., 2014b). Indeed, it has been suggested that alterations of magnocellular pathways may impact the coarse and fast visual analysis of emotional information. This hypothesis could be tested by exploring emotional processing of visual stimuli containing only low spatial (i.e., coarse information, mainly related to magnocellular pathways) or high spatial frequencies (i.e., detailed information, mainly related to parvocellular pathways; see D’Hondt et al., 2014b for more details). Moreover, future ERP studies should investigate the possible generalization of emotion decoding deficits to all kind of visual stimuli (e.g., facial expression, natural scene, posture) and sensorial modalities (e.g., prosody) as observed in AD (Maurage et al., 2009a).

### Systems Interactions Exploration

Future ERP should directly investigate reflective and automatic-affective systems interactions in AD and KS (Brion et al., 2015) to determine which stage(s) of information processing is (are) concerned by the disequilibrium between systems. To this end, gambling tasks such as the Iowa gambling task (Bechara et al., 1994) and the balloon analog risk task (Lejuez et al., 2002) may be relevant means. Using a gambling task, Kamarajan et al. (2010) found that reward processing was dysfunctional in AD by showing that AD individuals as compared with controls had significantly lower FRN and P3 amplitudes during loss and all outcome conditions, respectively. To go beyond the investigation of ERP components related to feedback processing, it would be interesting to use the version of the Iowa gambling task developed by Cui et al. (2013) to study ERP components related to choice evaluation and response selection. While we believe that this task would be useful, we also think that new strategies should be developed to more directly compare the impact of automatic and affective subcomponents on the reflective system. For instance, a Go/NoGo paradigm should be used in which inhibition occurs in a context where alcohol-related cues (for the automatic sub-system; see Petit et al., 2012 for a recent example) or affective stimuli (for the affective sub-system) are present (see **Figure 2**). ERP components related to inhibition (i.e., NoGo-N2 and -P3) and response monitoring could be therefore studied in the specific context of affective subsystem (emotional stimuli) or automatic subsystem (alcohol-related stimuli).

## Conclusion: Toward a Clinical Routine Involving ERP Technique

The main aim of this article was to stress the need for developing studies employing ERP, which have been up to now under-used in the domain, to obtain a clearer picture of KS impairments thanks to the high temporal resolution of this technique. We proposed new research avenues within the framework of dual-process models to better understand KS deficits regarding the reflective and automatic-affective systems, whose unbalanced interactions are supposed to be at the heart of AD.

According to the continuity hypothesis from AD to KS, and on the basis of ERP findings showing that activity of both systems is affected in AD, we can suppose: (1) a linear hypo-activation of both reflective system and affective subsystem; and (2) a graduated over-activation of the automatic subsystem. From a cognitive point of view, this assumption would lead to the deterioration from AD to KS of both executive functions and emotional decoding abilities and to a stronger bias toward alcohol-related stimuli. Alternatively, a linear worsening of brain impairments from AD to KS could also lead to a decreased activity in prefrontal and limbic areas and, therefore, a hypo-activation of both reflective and automatic-affective systems. This latter assumption calls into question the continuity hypothesis as it implies that deficits in KS do not result from a mere strengthening of deficits already present in AD. Instead, modifications in the activity of the automatic subsystem would be variable over the course of the disease, with an over-activation at the early stages (i.e., in AD) and then an under-activation at later stages (i.e., in KS; parallel to the decrease of craving, as after a long abstinence for instance). At this time, maintenance of the AD could be predominantly caused by an accentuation of the reflective system deficits. ERP investigations should help to examine the evolution of the two systems and their interactions during and between AD and KS. Moreover, further ERP studies should also investigate the possible role of a third system involving the insula as recently proposed by Noël et al. (2013) in their “triadic neurocognitive model of addiction.” According to this view, the insula, because of its role in the conscious representation of interoceptive signals, would mediate the impact of bodily changes associated with withdrawal, leading to the sensitization of the automatic-affective system and the reduction or the hijacking of the reflective system resources. The putative role of the insula in the imbalance between the automatic-affective and the reflective system needs, therefore, to be considered in both AD and KS.

Owing to its high temporal resolution, ERP technique should: (1) provide a refined diagnosis of KS impairments since, contrary to behavioral measures that give insights about the overall cognitive functioning, electrophysiological measures allow to identify modifications at each step of information processing: (2) help to identify early brain modifications in AD individuals at risk of developing neurological complication, before any detectable cognitive impairment at the behavioral level (see Maurage et al., 2009b for an illustration in binge drinking showing the usefulness of ERP in highlighting brain modifications while no behavioral deficits are observed). The evolution of ERP modifications could thus serve as a neurophysiological marker of KS vulnerability. This proposal is also in line with the recent call for biological markers that could be used in place of subjective clinical parameters currently employed in psychiatric diagnosis since ERP technique is already in full swing in psychiatry at the experimental and clinical levels (Campanella and Colin, 2014; McLoughlin et al., 2014). As recently proposed by Campanella (2013), a clinical routine involving ERP technique should be developed, allowing to refine the diagnosis of KS patients and to initiate individualized therapies, combining medication, psychotherapy and “ERP-oriented cognitive rehabilitation.” For instance, training AD response inhibition toward alcohol-related stimuli would promote abstinence (Houben et al., 2011), and, therefore, contribute to thwarting the high risk of patient drop out after detoxification (e.g., Finney et al., 1996; Fadardi and Cox, 2009). The relapse rate in AD may dwindle away with attentional-bias-reduction interventions since bias toward alcohol-related stimuli is correlated with the intensity of craving (Field and Cox, 2008; Field et al., 2009, 2013). Moreover, while behavioral improvements could be subtle, electrophysiological measures before and after rehabilitation would support clinical observation and serve as an indicator of treatment efficacy. Importantly, one possible limitation of the research avenues proposed here (see **Figure 2**) is that the massive cognitive deficits associated with KS would limit the ability of KS individuals to understand tasks instructions, to keep them in memory during the tasks, and to focus their attention during a sufficiently long period to complete the tasks. This explains why we tried to propose experimental procedures as simple as possible However, there are several lines of evidence in favor of the feasibility of our proposal: previous studies have been carried out with KS individuals using complex tasks [e.g., Stroop test, N-back paradigm, Trail making test, fluency, game of dice task, Brixton test (see Brion et al., 2014)]; there are evidence that KS individuals are capable of new learning (Kopelman et al., 2009); there are simple strategies to optimize the feasibility of these studies (such as breaks during the experiment allowing individuals to have a rest and to explain again task instructions)]. As illustrated in **Figure 2**, the perspectives proposed here to better understand electrophysiological correlates of emotion–cognition dysfunctions among KS patients could be ultimately used by clinicians as a tool to identify specific deficits, considering them as therapeutic targets to optimize patient’s quality of life, and ensure a follow-up measure of rehabilitation programs.

## Author Contributions

All authors listed, have made substantial, direct and intellectual contribution to the work, and approved it for publication.

## Conflict of Interest Statement

The authors declare that the research was conducted in the absence of any commercial or financial relationships that could be construed as a potential conflict of interest. The reviewer, Geraldine Petit, and handling Editor declared their shared affiliation, and the handling Editor states that the process nevertheless met the standards of a fair and objective review.
